# Modelling ultrasound-induced mild hyperthermia of hyperplasia in vascular grafts

**DOI:** 10.1186/1742-4682-8-42

**Published:** 2011-11-03

**Authors:** Mark R Brinton, Russell J Stewart, Alfred K Cheung, Douglas A Christensen, Yan-Ting E Shiu

**Affiliations:** 1 Department of Electrical and Computer Engineering, University of Utah, Salt Lake City, UT, USA; 2 Department of Bioengineering, University of Utah, Salt Lake City, UT, USA; 3 Department of Medicine, Division of Nephrology & Hypertension, University of Utah, Salt Lake City, UT, USA; 4 Medical Service, Veterans Affairs Salt Lake City Healthcare System, UT, USA; 5 Department of Electrical Engineering, Stanford University, 350 Serra Mall (Mail Code: 9505), Stanford, CA 94305-9505, USA

## Abstract

**Background:**

Expanded polytetrafluoroethylene (ePTFE) vascular grafts frequently develop occlusive neointimal hyperplasia as a result of myofibroblast over-growth, leading to graft failure. ePTFE exhibits higher ultrasound attenuation than native soft tissues. We modelled the selective absorption of ultrasound by ePTFE, and explored the feasibility of preventing hyperplasia in ePTFE grafts by ultrasound heating. Specifically, we simulated the temperature profiles of implanted grafts and nearby soft tissues and blood under ultrasound exposure. The goal was to determine whether ultrasound exposure of an ePTFE graft can generate temperatures sufficient to prevent cell growth on the graft without damaging nearby soft tissues and blood.

**Methods:**

Ultrasound beams from two transducers (1.5 and 3.2 MHz) were simulated in two graft/tissue models, with and without an intra-graft cellular layer mimicking hyperplasia, using the finite-difference time-domain (FDTD) method. The resulting power deposition patterns were used as a heat source for the Pennes bioheat equation in a COMSOL^® ^Multiphysics heat transfer model. 50°C is known to cause cell death and therefore the transducer powers were adjusted to produce a 13°C temperature rise from 37°C in the ePTFE.

**Results:**

Simulations showed that both the frequency of the transducers and the presence of hyperplasia significantly affect the power deposition patterns and subsequent temperature profiles on the grafts and nearby tissues. While neither transducer significantly raised the temperature of the blood, the 1.5-MHz transducer was less focused and heated larger volumes of the graft and nearby soft tissues than the 3.2-MHz transducer. The presence of hyperplasia had little effect on the blood's temperature, but further increased the temperature of the graft and nearby soft tissues in response to either transducer. Skin cooling and blood flow play a significant role in preventing overheating of the native tissues.

**Conclusions:**

Modelling shows that ultrasound can selectively heat ePTFE grafts and produce temperatures that cause cell death on the graft. The temperature increase in blood is negligible and that in the adjacent soft tissues may be minimized by skin cooling and using appropriate transducers. Therefore, ultrasound heating may have the potential to reduce neointimal hyperplasia and failure of ePTFE vascular grafts.

## Background

There are two primary types of permanent hemodialysis accesses, native arteriovenous (AV) fistulae and expanded-polytetrafluoroethylene (ePTFE) AV grafts. When compared to the fistula, AV grafts provide vascular access with higher blood flow rates, do not require a maturation period, and can be used sooner after surgical placement. Grafts are a particularly useful form of vascular access in patients whose vasculature is insufficient to support a functional native AV fistula. The latter is important because up to 60% of AV fistulae in the United States fail to mature and become functional. Despite these advantages, only 23.3% of the permanent hemodialysis vascular accesses in the United States are AV grafts [1], largely due to their low one- and two-year primary patency rates, being 50% and 25%, respectively [2,3].

The low primary patency rates of ePTFE grafts are attributed to their propensity to develop excessive cell growth in the graft lumen, preferentially near the graft-venous anastomosis site [4]. This growth, termed neointimal hyperplasia (NH), causes lumen stenosis that predisposes to thrombosis and occlusion. If the occurrence of NH could be prevented or reduced, the synthetic graft should be an excellent form of dialysis vascular access for the large population of dialysis patients whose vasculature cannot support a functional AV fistula.

Strategies to prevent or reduce NH in synthetic AV grafts include the use of drug-eluting stents [5,6], endothelialization of the graft lumen [7-9], antiproliferative drugs delivered using hydrogel systems to prevent cell overgrowth [5,6,10-12], and catheter-based gamma radiation [13]. The stent and catheter treatments require invasive procedures, and none of the strategies has proven effective in humans.

Ultrasound has been used for a variety of therapeutic applications including muscle diathermy and bone healing [14], drug delivery [15] and mild hyperthermia [16-18]. Ultrasound exposure has been shown to reduce smooth muscle cell proliferation *in vitro *[19]. Intravascular ultrasound has also been shown to reduce NH in implanted stents by 35% [20]. Ultrasound is comprised of acoustic or vibrational waves that carry mechanical energy. The amount of acoustic energy that is deposited in a material and converted into heat is approximately proportional to the material's viscosity [21]. Since ePTFE exhibits 5 to 10 times more ultrasound attenuation (absorption and scattering) than native soft tissues, transcutaneous delivery of ultrasound may provide a noninvasive method to selectively heat an implanted ePTFE graft, thereby preventing cell growth and NH on the graft surface [22,23].

In this study, we modelled the selective absorption of ultrasound in ePTFE and the corresponding thermal profiles to determine *whether ultrasound exposure can elevate the graft to temperatures capable of inflicting NH cell death without significantly damaging the nearby tissues and blood*. Specifically, we used mathematical and computational modelling to simulate the ultrasound power absorbed and the effectual temperature profiles in implanted grafts and nearby tissues and blood under ultrasound generated from two different transducers.

## Methods

### Overview

The modelling involved two steps. In the first step, the power deposited throughout a simplified ePTFE graft/tissue model was calculated using a three-dimensional (3D) finite-difference time-domain (FDTD) technique [24-27]. A main advantage of the FDTD method is its ability to handle heterogeneous models readily. The power deposition values, serving as the heat source, were next used to generate 3D temperature profiles using COMSOL^® ^Multiphysics software and the Pennes bioheat equation [28]. Two transducers with different frequencies, focal lengths and diameters (Table 1) were used to generate power deposition patterns. Since 50°C is known to cause cell death [29], the transducer powers were adjusted to produce a 13°C temperature increase from 37°C in the ePTFE. The temperatures after 5-second or 30-second ultrasound exposures, as well as during the cooling period back to 37°C, were also modelled.

**Table 1:** Transducer parameters.

	*Transducer 1*	*Transducer 2*
**Frequency**	1.5 MHz	3.2 MHz

**Focal length**	18 cm	3.5 cm

**Diameter**	10 cm	2.5 cm

**Acoustic power**	0.6 W	0.375 W

### FDTD Simulations

To obtain a 3D pattern of the ultrasound power density deposited in the graft/tissue models, we applied the FDTD numerical technique to equations that describe the propagation of an acoustic pressure wave through a medium. The 3D models have a form composed of individual regularly spaced cuboidal voxels, each with possibly different densities, speeds of sound and attenuation properties, and with planar front faces. Acoustic waves can be described using Newton's force equation and the conservation of mass. Using finite differences these equations can be solved for the acoustic pressure and the particle velocity in the x, y and z directions. The method of finite differences involves replacing classical derivatives with discrete differences in time and on a 3D grid space. For example, the derivative of the *x*-direction particle velocity with respect to *x *becomes the difference of the particle velocity at locations *x *and *x + Δx *divided by *Δx*, the FDTD grid resolution in the *x *direction (see details in Appendix A). Using initial pressure values, the particle velocities in the *x*, *y *and *z *directions for the entire grid and the first time step are calculated. Then, using the newly calculated particle velocities, new pressure values for the same time step are calculated. Continuing in this manner the program, written in MATLAB, works by iterating back and forth between space and time steps solving for the particle velocities and pressure as the acoustic wave propagates through the 3D gridded models of the inhomogeneous medium.

**Figure 1:**
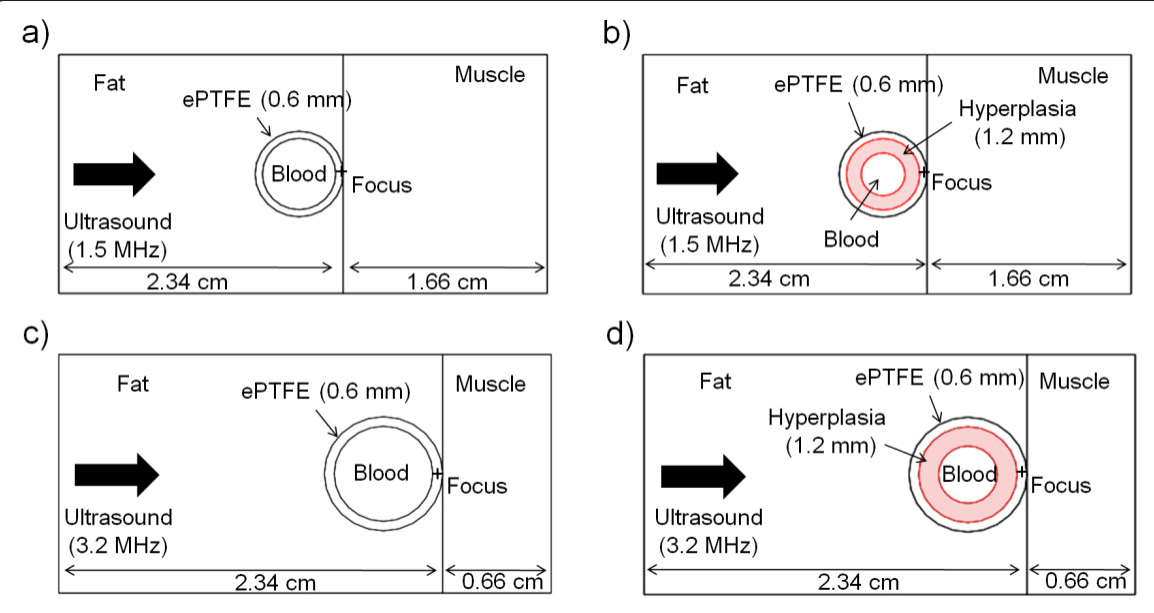
**Slice views of the 1.5-MHz and 3.2-MHz transducer models with and without hyperplasia**. Slice views transverse to the graft at the center of the 1.5-MHz transducer model (a) without hyperplasia and (b) with hyperplasia; (c) and (d) show the same slices for the 3.2-MHz transducer model. The scale is not the same for each model; the 1.5-MHz model dimensions are 2 × 2 × 4 cm, while the 3.2-MHz model is 1.5 × 1.5 × 3 cm. In both models the graft is cylindrical with a 0.6-cm inner diameter and a 0.6-mm wall thickness. The skin surface is located at the left boundary of each figure.

Since the two transducers that were modelled had spherically curved emitting surfaces as described in Table 1 we needed to first propagate the pressure waves in the water region between the curved transducer surfaces and the front plane of the model (corresponding to the skin surface). This stand-off distance was 15.66 cm and 1.16 cm for the 1.5-MHz and 3.2-MHz transducers, respectively. Instead of employing the FDTD method for this step, we used the Rayleigh-Sommerfeld integral approach [30] (appropriate for a homogeneous medium such as water) to find the pressure pattern on the front model plane, which then became the incident pressure pattern for the FDTD calculations. Four simplified ePTFE graft/tissue models (see Figure 1) with surrounding muscle, fat and internal blood, were created in MATLAB, two for each of the two transducers analyzed. No special FDTD boundary conditions were found to be necessary; the FDTD pressure solution was terminated before the beam reflected off the back wall of the large grid. Reflections from the four side walls of the model were insignificant because the beam cross-section was shrinking due to focusing.

The accuracy of the FDTD method depends on the chosen temporal and spatial step size. While smaller step sizes more closely approximate the original differential equations and tissue geometry, small step sizes result in large models and long calculation times. FDTD simulations were completed on a computer using an Intel^® ^Core™ i7 processor at 2.67 GHz and with 11.9 GB RAM. To avoid aliasing, the model grid spacings Δ*x*, Δ*y *and Δ*z *were set to 0.1 mm for the 1.5-MHz models and to 0.05 mm for the 3.2-MHz models, roughly 1/10^th ^of the acoustic wavelength in water for the respective frequencies. The overall dimensions of the 1.5-MHz models were 2 × 2 × 4 cm, producing a model with 200 × 200 × 400 elements. Because the 0.05-mm step size in the 3.2-MHz models would produce eight times as many elements as the 1.5-MHz models (exceeding the memory limits of the computer), the overall dimensions of the 3.2-MHz model were reduced to 1.5 × 1.5 × 3 cm, resulting in 300 × 300 × 600 elements. Some clipping of the incident power occurred since the areas of the front planes of the models (1.5 × 1.5 cm and 2 × 2 cm) were slightly smaller than the extent of the beams from the transducer. This clipping caused 2.5% and 7.5% reductions in the power from the 1.5- and 3.2-MHz transducers, respectively.

### Physiological models for acoustic simulation

Physiological acoustic properties (Table 2) [31,32] were used in the FDTD calculations. Two scenarios were modeled for each transducer: grafts free of NH and grafts with 1.2-mm thick hyperplasia. Even though NH most commonly occurs at the graft-vein anastomosis, for simplicity the model did not include the anastamosis and modeled NH in a straight ePTFE tube. This simplified model effectively modeled selective ultrasound power deposition and heating in the graft, as well as conductive and convective heat transfer.

**Table 2:** Material properties used for acoustic FDTD and COMSOL thermal modelling.

	*Blood*	*Muscle*	*Fat*	*ePTFE*
**Density (*ρ*) [kg/m^3^]**	1060^b^	1070^b^	937^b^	1230^c^

**Specific heat capacity (*Cp*) [J/kg·K]**	3894^a^	3140^a^	2600^a^	1172^d^

**Thermal conductivity (*k*) [W/m·K]**	0.5^a^	0.5^a^	0.7^a^	0.35^d^

**Perfusion (*ω*) [s^-1^]**	NA	6.776e-4^a^	4.373e-4^a^	NA

**Speed of sound (*c*) [m/s]**	1580^b^	1579^b^	1426^b^	1237^c^

**Attenuation (α) at 1.5/3.2 MHz [Np/m]**	4.5/9.6^b^	13.5/29.7^b^	10.5/22.4^b^	116/247.6^c^

Obtained from ^a^Duck [31], ^b^Christensen [21], ^c^Vickers [23] and ^d^Incropera [32].

Using the FDTD algorithm, the acoustic pressure wave was calculated as it moved through the *x*, *y *and *z *grid space. The absorbed power density *Q*, in W/m^3^, is the time average (within each cycle) of power deposited per unit volume throughout the 3D model. It depends on the acoustic pressure and the material through which the wave is travelling as follows [21]:

(1) Q=α|p2|ρc(1+(α⋅c)2(2π⋅f)2),

where *α *is material pressure attenuation coefficient, *ρ *is material mass density, *c *is speed of sound, *p *is the rms pressure and *f *is the frequency of the wave. During the duration of the ultrasound exposure, the magnitude of the applied acoustic power and pressure was held constant. After calculating *Q *from the 3D FDTD pressure solution, it was used as a heat source in the Pennes bioheat equation.

### Heat transfer using COMSOL Multiphysics

The general 3D heat transfer application in COMSOL Multiphysics, a commercial finite-element method (FEM) modelling software designed to solve heat transfer, acoustic, fluid flow and other engineering problems described by differential equations, was used. The simplified graft/tissue models used for the FDTD calculations were also used in COMSOL. The thermal properties assumed for the blood, ePTFE, muscle and fat regions are listed in Table 2.

To simulate skin cooling, the front fat boundary was held constant at 20°C. Except where the blood entered the graft/tissue model (where the entering blood temperature was held constant at body temperature of 37°C), all other exterior boundary conditions were considered insulated, allowing no heat flux across exterior boundaries. Insulated boundaries provide an accurate assumption if the simulated volume is large enough that the side and rear boundaries are much farther from the ultrasound heated region than the nominal diffusion length.

Physiologically relevant blood flow volumes and velocities were included in the modelling, and the COMSOL Multiphysics software has the capability to model flow within the heat transfer application. In implanted grafts, blood flow near the anastomosis is often turbulent, and may contribute to neointimal proliferation [33]. However, for simplicity, convection in the graft lumen was modelled using laminar blood flow. Volumetric flow rates based on clinical data were used to determine the peak and mean velocities for the laminar velocity distribution. For the graft without NH, given a luminal cross-sectional area of 28.3 mm^2^, an average flow rate of 412 ml/min was assumed [4], resulting in an average flow velocity of 0.243 m/s. Since grafts exhibiting flow rates less than 100 ml/min are considered failing, for the 1.2-mm thick NH model this flow rate was used to calculate an average flow velocity of 0.164 m/s. From the average flow velocities the laminar flow profiles were defined and implemented in the COMSOL models.

The simulated heat transfer equation was the Pennes bioheat equation:

(2) $$\begin{array}{c} \rho \cdot Cp\frac{\partial T}{\partial t}=\nabla \cdot k\nabla T+Q+Q_{perf}\\ +A, \end{array}$$

where *ρ *is mass density [kg/m^3^], *k *is thermal conductivity [W/m·K], *T *is temperature [K], *A *is a metabolic heat generation term [W/m^3^] (which was neglected because it was small compared to acoustic power deposition *Q *[W/m^3^]), *Cp *is specific heat capacity [J/kg·K], and *Q_perf _*represents heat transfer loss due to perfusion [W/m^3^]. Because the general heat transfer application in COMSOL does not include the thermal effects of perfusion included in the Pennes bioheat equation, the effects of perfusion were simulated in the muscle and fat tissues using

(3) $$\begin{array}{c} Q_{perf}=\left(T_{blood}-T\right)\times \left(1-\kappa \right)\\ \times \rho_{blood}\times Cp_{blood}\times \omega_{tissue}, \end{array}$$

where *ω *is the blood perfusion rate of the tissue [s^-1^] and κ is a term to account for the incomplete thermal transfer to perfused blood. In our simulations κ was assumed to be zero (also assumed by Pennes and most subsequent investigators [34]) to model complete thermal equilibration between the blood and tissue.

Thermal damage to healthy native tissues, a major concern during hyperthermia, can be reduced with active skin surface cooling [35,36]. The pre-exposure skin cooling was simulated in COMSOL by solving a steady-state model prior to ultrasound exposure. In this model, the skin boundary was held constant at 20°C and the blood entering the graft and perfusing the tissue was held constant at 37°C. Using the skin-cooling model, the temperatures resulting from 10-second or 30-second transient applications with the ultrasound power deposition values from the FDTD simulations were calculated. To explore the effects of intermittent ultrasound exposure, the temperatures during the cool-down period following the ultrasound exposure were simulated.

## Results

### Simulated pressure and power depositions for the 1.5- and 3.2-MHz transducers

**Figure 2:**
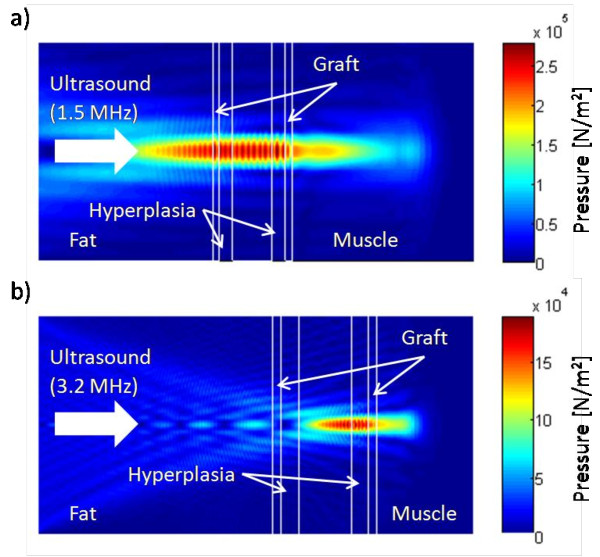
**Pressure magnitude in the central plane aligned with the graft length from the FDTD model**. (a) The focal zone of pressure produced by the 1.5-MHz transducer extends across the graft. In contrast, (b) the focal zone of pressure produced by the 3.2-MHz transducer covers only the rear graft wall. Intimal hyperplasia models are shown. Both transducers were focused on the rear graft wall.

Our simulated pressure results showed that, while the focus was positioned at the rear graft wall for both transducers, the focal zone of the 1.5-MHz transducer spanned the width of the graft (Figure 2a) while the focal zone of the 3.2-MHz transducer covered only the rear graft wall (Figure 2b). Reflection and absorption of the ultrasound beam by the ePTFE graft can be seen in the reduction of the pressure magnitude behind the rear ePTFE wall, at which location the magnitudes of the pressures from the 1.5- and 3.2-MHz transducers were reduced to about 2.0 × 10^5 ^N/m^2 ^and 1.2 × 10^5 ^N/m^2^, respectively.

**Figure 3:**
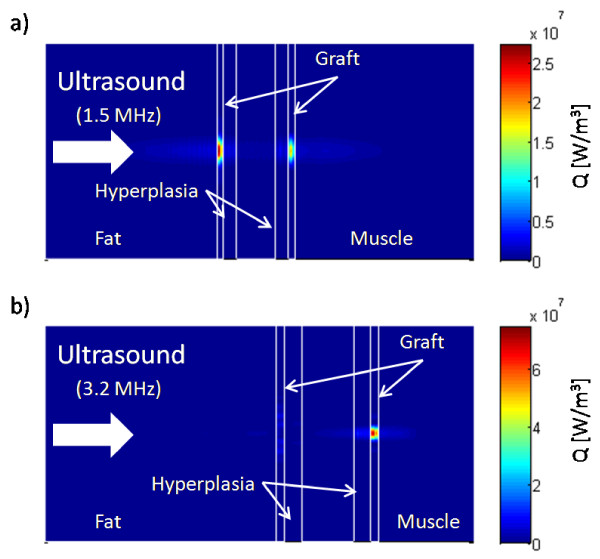
**Power deposition Q in the central plane aligned with the graft length from the FDTD model**. Q in the central plane aligned with the graft length was calculated for the (a) 1.5-MHz transducer (0.6 W incident power) and (b) 3.2-MHz transducer (0.375 W incident power), focused on the back wall. Intimal hyperplasia models are shown. With the 1.5-MHz transducer, the ePTFE absorbed more power at the front wall, leaving less to be absorbed in the rear graft wall, while the 3.2-MHz transducer deposited most of its power in the rear wall of the graft.

The high acoustic attenuation of ePTFE caused approximately five times more power to be deposited within the graft than the adjacent tissues (Figure 3). The maximum *Q *for the 1.5-MHz transducer was 2.75 × 10^7 ^W/m^3 ^in the front graft wall (Figure 3a), and for the 3.2-MHz transducer, almost 8 × 10^7 ^W/m^3 ^(Figure 3b) in the rear graft wall.

### Simulated temperature profiles

**Figure 4:**
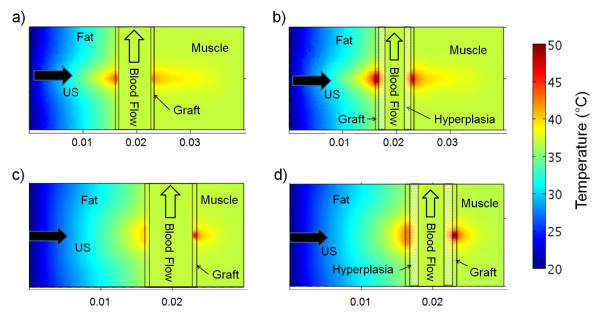
**Temperature in the graft models, in the central plane aligned with the graft length, after 30 seconds of ultrasound exposure**. 0.6 W of acoustic power was applied to the 1.5-MHz models with (a) no hyperplasia and (b) 1.2-mm of hyperplasia. 0.375 W was applied to the 3.2-MHz models with (c) no hyperplasia and (d) 1.2-mm of hyperplasia. With hyperplasia, 50°C was reached within the graft material for both transducers.

**Figure 5:**
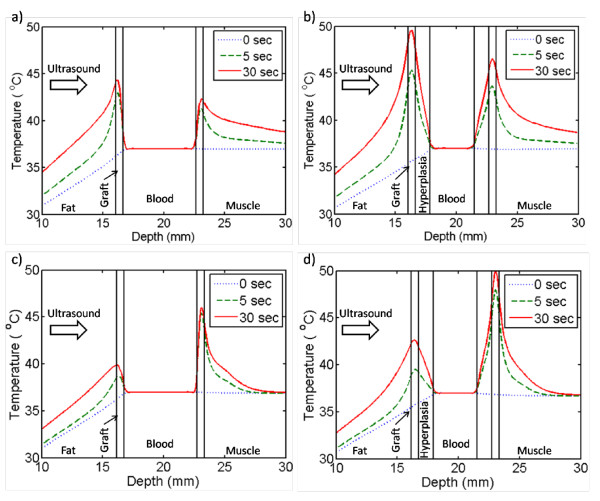
**Temperatures along the direction of ultrasound propagation, through the center of the ultrasound focal zone**. Temperature along the direction of ultrasound propagation through the center of the ultrasound focal zone after 0, 5, and 30 seconds of ultrasound exposure. The 1.5-MHz transducer models are shown in (a) and (b). The 3.2-MHz transducer models are shown in (c) and (d). After 30 seconds, the simulations showed a temperature of approximately 50°C in the graft walls for models (b) and (d) with 1.2-mm thick intimal hyperplasia.

The temperatures in the center 2D slice in line with the length of the graft after 30 seconds of ultrasound exposure are shown in Figure 4. Tissue and graft temperatures along the direction of ultrasound propagation through the center of the ultrasound focus are plotted in Figure 5. Modelling of NH-free grafts showed that the 1.5-MHz transducer caused more heating at the front graft wall (Figures 4a and 5a) while the 3.2-MHz transducer generated more heat at the rear graft wall (Figures 4c and 5c); the maximal temperature reached in either of the graft wall locations was 45°C for both transducers for the case of NH-free grafts. Heat convection due to blood flow acted as a large heat sink. However, with hyperplasia the convective boundary was moved 1.2 mm away from the ePTFE wall. Consequently, in the models with hyperplasia, both transducers caused graft-wall heating up to 50°C. Note that the 3.2-MHz transducer heated less adjacent soft tissue than the 1.5-MHz transducer; the 40°C region extended about twice as deep into the muscle tissue for the 1.5-MHz exposure (Figure 4b) compared to the 3.2-MHz results (Figure 4d). Both transducer models predicted very little heating in the blood, even with the reduction in volumetric flow from 412 ml/min (in the absence of hyperplasia) to 100 ml/min (in the presence of hyperplasia).

**Figure 6:**
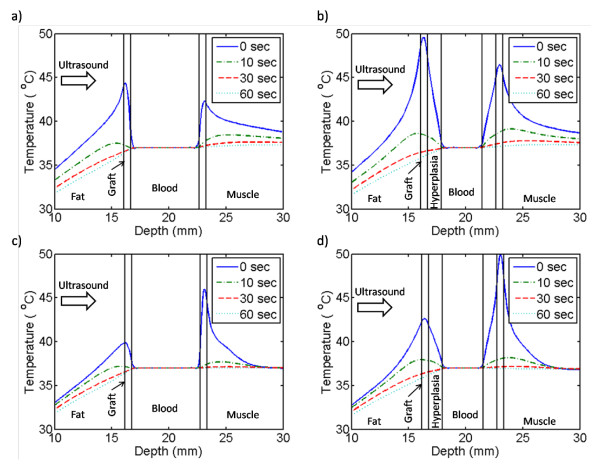
**Post ultrasound-exposure cooling profiles**. Post ultrasound-exposure cooling profiles. Temperatures are along the direction of ultrasound propagation through the center of the ultrasound focus at times 0, 10, 30 and 60 seconds after termination of exposure. The 1.5-MHz transducer models are shown in (a) and (b). The 3.2-MHz transducer models are shown in (c) and (d). After 30 seconds, temperatures cool to within 1°C of 37°C (body temperature).

Figure 6 shows the temperature profiles immediately after a 30-second ultrasound exposure. Ten seconds after the exposure from the 1.5-MHz transducer had ceased, the temperatures cooled to within 2 and 2.5°C of body temperature (37°C) for the hyperplasia-free and hyperplastic grafts, respectively. During the same 10-second duration, grafts that were exposed to the 3.2-MHz transducer cooled faster and their temperatures were within 1 and 2°C of body temperature for the hyperplasia-free and hyperplasic grafts, respectively. Sixty seconds after the ultrasound exposure ceased, graft temperatures had returned to the pre-exposure values under all conditions (i.e., for both frequencies and with or without hyperplasia).

## Discussion

### 1.5- and 3.2-MHz transducer modelling

Wave frequency is an important parameter in therapeutic ultrasound. Ultrasound attenuation increases with frequency; consequently, high-frequency ultrasound is used for imaging and therapy near or at the body surface, while low-frequency ultrasound is appropriate for deeper imaging and therapy [37,38]. Frequency also has an effect on the dimensions of the focal zone. Higher frequency waves focus to smaller zones [21]. The increase in frequency from 1.5 MHz to 3.2 MHz, a factor of 2.1, causes the focal length and width to decrease by this same factor for a given transducer geometry. In addition, the difference in the ratio of transducer focal length to diameter for the 1.5- and 3.2-MHz transducers, 1.8 and 1.4, respectively, further reduces the length of the focal zone by a factor of 1.3. These differences are evident in Figure 2. The focal zone for the 3.2-MHz transducer was small enough that power was deposited primarily in the rear graft wall (Figure 3b).

The amount of ultrasound power absorbed by a material depends on, among other things, the material's acoustic attenuation (Equation 1). The attenuation of ePTFE is almost 10 times greater than the tissues surrounding the ePTFE graft (Table 2). Consequently, the ePTFE material absorbed much more power than native tissue from the ultrasound beam. This strategic concentration of power created the high temperatures within the graft and the mild heating in the surrounding tissues (Figures 4 and 5).

The higher frequency and the smaller focal zone (resulting in higher beam intensity) of the 3.2-MHz transducer caused a higher, more concentrated power deposition pattern. Although the 3.2-MHz transducer operated at 63% of the power of the 1.5-MHz transducer it deposited a maximum *Q *approximately three times more than the 1.5-MHz transducer (Figures 3a and 3b). Interestingly, the 3.2-MHz transducer generated similar temperatures while depositing a higher maximum *Q *with less overall power than the 1.5-MHz transducer. The roughly three-fold *Q *of the 3.2-MHz transducer induced a faster but more localized increase in temperature than the 1.5-MHz transducer. As a result, the more localized heating produced larger spatial temperature gradients. These large gradients gave rise to greater heat transfer away from the focal region of the 3.2-MHz transducer, limiting graft and tissue temperatures to be similar to those in the 1.5-MHz transducer model after 30 seconds of exposure.

### Temperature modelling

Understanding cellular response to specific temperatures provides relevance for the thermal modelling. While cellular response to thermal exposure varies with cell species and origin [39], both apoptosis (organized cell death) and necrosis (ruptured cellular membrane) can occur in response to thermal exposure. In general, temperatures below 43°C can cause a heat-shock-protein response but only induce apoptosis mildly [40,41]. Samali et al. [42] and Harmon et al. [43] exposed suspended cells for one hour and for 30 minutes, respectively, and concluded the turning point from apoptosis to necrosis occurred at 45°C. Apoptosis, rather than necrosis, is the preferred cell death pathway for our target clinical application. Necrotic death causes inflammation and the attraction of macrophages that may exacerbate NH formation. Hehrlein et al. reported that vascular damage from high-temperature exposure led to increased intimal growth in canines [44]. In addition, temperatures exceeding 47°C cause epidermal and dermal injury [45]. Taken together, the target graft temperature should be between 45-47°C to induce apoptotic death while limiting necrotic death.

Differences in thermal exposure between the 1.5- and 3.2-MHz transducer simulations may influence the extent of cell death on implanted ePTFE grafts. For example, the 3.2-MHz transducer heated the graft to a higher temperature after 5 seconds (Figures 5b and 5d). Consequently, using the 3.2-MHz transducer the luminal graft surface may reach effective elevated temperatures more quickly, thereby heating hyperplasia at effective temperatures for a greater portion of the 30 second ultrasound pulse. The 3.2-MHz transducer modelling also showed less native tissue heating above 43°C. As a result, the 3.2-MHz transducer's more confined spatial heating may facilitate thermal exposure at the anastomosis site with less damage to adjacent tissues, which is a major advantage when compared to the 1.5-MHz transducer.

Our modelling results suggest that the ultrasound should be delivered in an intermittent manner in our target clinical application. Non-continuous ultrasound therapy helps to reduce unwanted heating of healthy tissues by allowing cooling between the ultrasound pulses. Pulsed ultrasound exposure could also help ensure that hyperplasia receives only mild heat, thereby avoiding tissue necrosis. To avoid residual temperature buildup during pulsed ultrasound exposure, a 30-second exposure should be followed by a 30-second rest, for a duty cycle of 0.5. Shorter rest times would not allow sufficient time for the tissue to return to 37°C before the next ultrasound pulse exposed the ePTFE graft. Consequently, a treatment equivalent to 30 minutes at 45°C would take twice as long, lasting up to an hour.

During the ultrasound exposure the low thermal conductivity of the tissue slowed thermal diffusion into the tissue. Thus, after 30 seconds of exposure, the rise in temperature was confined to and in close proximity of the ePTFE material (Figure 5). Furthermore, the heat that did transfer into the tissue also dissipated slowly during the cooling cycle, evident by the regions in the fat and muscle which cooled to the pre-exposure temperatures last (Figure 6). Consequently, in our target clinical application the cooling time between consecutive ultrasound pulses, which limited by the thermal conductivity of the tissue, is necessary in order to avoid thermal buildup.

Due to convection, the blood-ePTFE boundary (Figures 5a and 5c) and the blood-NH boundary (Figures 5b and 5d) did not reach temperatures capable of inducing apoptotic cell death, suggesting ultrasound heating may not prevent NH formation in NH-free grafts and may not be able to treat pre-existent NH entirely, respectively. Attempts to heat more of the NH region using longer ultrasound exposures could cause thermal damage to tissue adjacent to the graft where the heat dissipated more slowly. However, temperatures capable of inducing apoptosis were generated near the NH-ePTFE boundary (Figures 5b and 5d). As a result, repeated ultrasound exposure may reduce NH growth and delay the development of significant stenosis. In the presence of NH, a graft can still remain functional as long as the NH size is not so big that it impedes blood flow. Therefore, even though the proposed approach may not completely prevent the formation of NH or remove a pre-existing NH entirely, repeated ultrasound exposure may restrict the NH size, thereby lengthening the patency rate of the graft.

## Conclusions

Results of our modelling showed selective ePTFE graft heating could be achieved by either the 1.5-MHz or the 3.2-MHz transducer. Both transducers can lead to temperatures capable of inducing apoptosis near the NH-ePTFE boundary. The temperature increase in blood was negligible using either transducer, while the temperature increase in the adjacent soft tissue was smaller when the 3.2-MHz transducer was used. Tissue temperature variations due to the presence or absence of NH suggest the need of patient-specific ultrasound exposure protocols. Overall, intermittent ultrasound heating may have the potential for clinical application to reduce neointimal hyperplasia and failure of ePTFE vascular grafts.

## Competing interests

The authors declare that they have no competing interests.

## Authors' contributions

MRB carried out the modelling and drafted the manuscript. RJS, DAC and YES conceived of the study. AKC, DAC and YES participated in the design of the study and helped to draft the manuscript. All authors read and approved the final manuscript.

## Appendix A

### Detailed FDTD method

Acoustic waves can be described [46] using Newton's force equation (A1) and the conservation of mass (A2):

(A1) $$\frac{\partial ū}{\partial t}=\frac{\nabla p}{\rho_{o}}+\frac{\xi }{\rho_{o}}\nabla^{2}ū$$

(A2) $$\frac{\partial p}{\partial t}=-\frac{1}{K}\nabla \cdot ū,$$

where

*ū *= *u_x _ẋ + u_y _ẏ + u_z _ż *is the particle velocity,

*u_x_*, *u_y_*, and *u_z _*are the respective particle velocities in the *ẋ*, *ẏ*, and *ż *directions,

*p *is the pressure,

$$\xi =\left(\eta +\frac{4}{3}\eta \text{’}\right),$$

*η *is the dynamic coefficient of shear viscosity of the material,

*η*' is the dynamic coefficient of bulk viscosity of the material,

*ρ_o _*is the average mass density of the material, and

*Κ *is the compressibility of the material.

Considering compression waves only (shear waves attenuate rapidly with distance [46]), these two equations can be solved in 3D using an FDTD method. Solving for pressure *p *in finite-difference form, (A2) yields

(A3) $$\begin{array}{c} p_{ijk}^{n+1}=-\frac{\Delta t}{K_{ijk}}\left(\frac{u_{x_{i+1jk}}^{n}-u_{x_{ijk}}^{n}}{\Delta x}+\frac{u_{y_{ij+1k}}^{n}-u_{y_{ijk}}^{n}}{\Delta y}\right)\\ \left(+\frac{u_{z_{ijk+1}}^{n}-u_{{}_{z_{ijk}}}^{n}}{\Delta z}\right)+p_{ijk}^{n}. \end{array}$$

**Figure 7:**
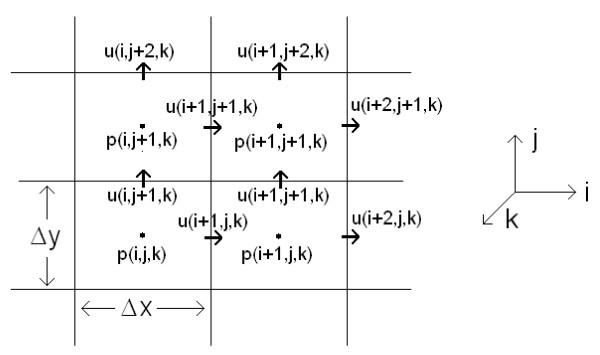
**FDTD grid space to solve Equations (A1) and (A2)**. FDTD grid space to solve Equations (A1) and (A2). This figure shows pressure and velocity in two dimensions only. The third dimension, z, is perpendicular to the x-y plane.

Here Δ*x*, Δ*y*, and Δ*z *are the lengths of the voxels in the computational grid space, the letters *i*, *j*, and *k *denote the *x*, *y *and *z *indices, respectively (see Figure 7), and Δ*t *is the increment of time between steps. For convergence, Δ*t *is limited by the Courant condition [24]. The superscript *n *denotes the current time step, and *n+1 *represents the next time step. The *u_x _*component of the velocity from (A1) is given in finite-difference form in (A4). The *u_y _*and *u_z _*solutions (not shown) are similarly defined.

(A4) $$\begin{array}{c} u_{x_{ijk}}^{n+1}=\frac{-\Delta t}{\rho_{ijk}^{\prime }\Delta x}\left(p_{ijk}^{n}-p_{i-1jk}^{n}\right)+u_{x_{ijk}}^{n}+\frac{\Delta t}{\rho_{ijk}^{\prime }\Delta x^{2}}\left[\xi_{ijk}{u_{x}}_{x_{i+1jk}}^{n}\right)\\ +\xi_{i-1jk}u_{{}^{x_{i-1jk}}}^{n}-\left(\xi_{ijk}+\xi_{i-1jk}\right)\cdot u_{x_{ijk}}^{n}. \end{array}$$

Because the velocity *u *is defined on the boundaries of each voxel, the material density *ρ*' also must be defined on the edges. As a result, *ρ*' is approximated as the average density of two adjacent cells:

(A5) ρ'ijk=(ρi−1jkn+ρijkn)2.

The FDTD algorithm works iteratively between (A3) and (A4), as well as between the respective equations for the *u_y _*and *u_z _*velocity solutions not shown here, to calculate the pressure and velocities at each time step throughout the entire model grid space.
